# Serological and Community Awareness Study of Lumpy Skin Disease in Different Agro-Ecological Zones of Sidama Regional State, Southern Ethiopia

**DOI:** 10.3390/ani14121782

**Published:** 2024-06-13

**Authors:** Nebyou Moje, Adane Seifu, Gizachew Hailegebreal, Dereje Shegu, Serena Montagnaro, Gianmarco Ferrara

**Affiliations:** 1 College of Veterinary Medicine and Agriculture, Addis Ababa University, Bishoftu P.O. Box 34, Ethiopia; 2 Faculty of Veterinary Medicine, Hawassa University, Hawassa P.O. Box 05, Ethiopia; adeseifuhotessa@gmail.com (A.S.); gizachew05@gmail.com (G.H.); 3 Animal Health Institute (AHI), Sebeta P.O. Box 04, Ethiopia; dshegu@yahoo.com; 4 Department of Infectious Diseases, University of Naples, 80138 Naples, Italy; serena.montagnaro@unina.it (S.M.); gianmarco.ferrara@unina.it (G.F.)

**Keywords:** agroecology, LSD, LSD outbreak, sero-epidemiology, risk factor, qualitative study

## Abstract

**Simple Summary:**

Lumpy skin disease (LSD) is a preventable disease in cattle. Understanding the epidemiology of the disease, as well as farmers’ perceptions, is the most important key to designing a prevention strategy. Accordingly, the current study was designed to establish LSD seroprevalence using specific methods and to investigate LSD epidemiology in the Sidama regional state (Ethiopia), as well as to assess farmers’ perceptions. The study determined high exposures to LSD at both herd and animal levels in three agro-ecological zones. Furthermore, the farmers’ knowledge about LSD was low, and there were complaints of LSD disease reports even in vaccinated groups. As a conclusion, this study identified the distribution of LSD in different management systems as well as agro-ecological zones, followed by a low level of farmers’ knowledge about the disease. Furthermore, this study indicates the necessity for community-based awareness campaigns regarding clinical signs of LSD along with regularly updated information on LSD prevalence.

**Abstract:**

The lumpy skin disease (LSD) vaccination status and epidemiological distribution remain unknown in some parts of Ethiopia, including the Sidama regional state. In this study, a serological survey of LSD was performed using a specific virus neutralization assay in selected districts of the Sidama regional state representing three agroecological zones from September 2021 to June 2022. Moreover, an assessment of community awareness and LSD vaccine-related problems was conducted using a questionnaire. Our results showed an overall animal and herd level seroprevalence of 40.8% (95%CI = 35.8, 45.8) and 81% (95%CI = 77, 85), respectively. High and low seroprevalence were observed in lowland (48%) and highland (28%) areas, although they were not statistically significant. However, risk factors such as management systems and breeds showed substantial differences in their LSD prevalence. The results obtained through the questionnaire showed that a small portion of respondents (29.2%) know about LSD and vaccinate their cattle (23.3%) against this disease. Only 20.8% of the respondents stated that there was LSD occurrence in their vaccinated cattle. In conclusion, both qualitative and quantitative study results showed the need for intervention in terms of community-based awareness creation about LSD clinical signs and vaccination advantages together with the frequently updated information on LSD prevalence.

## 1. Introduction

Livestock farmers in Ethiopia keep cattle for multiple purposes, such as milk production, draft power, beef production, and manure for fuel and fertilizers [1]. Among all the livestock that constitute Ethiopia’s farm animals, ruminants (cattle, sheep and goats) are the most dominant livestock species and have socio-economic benefits [2].

In developing countries, such as eastern Africa, the livestock production system is increasingly affected by the competition for natural resources, specifically land and water [3]. Livestock diseases are the other factors that can affect the growth of the livestock population. From livestock diseases, lumpy skin disease (LSD) stands among the major diseases that limit the productivity of cattle, having huge economic impacts in different parts of Ethiopia [4,5].

Lumpy skin disease was believed to have been introduced in Ethiopia for the first time through the northwest in 1981 [6]. After the introduction, the disease initially spread eastward, then later to all directions; currently, it has affected all regions and agro-climatic zones of the country. Communal grazing and watering, uncontrolled cattle movements, and pastoralism can be mentioned as ways to enhance the spread of LSD. The poor animal health situation, inefficient prevention, and control efforts, in combination with late detection of the disease, have further contributed to spreading LSD in Ethiopia. This means a lot to the more than 65 million estimated cattle population of this country, causing a huge socio-economic impact [7,8].

Lumpy skin disease is a severe viral disease in cattle that often occurs as regional epidemics within a larger area in which it is endemic [4,9]. It is caused by lumpy skin disease virus (LSDV), which is a member of *Capripoxvirus* (CaPVs) genus with large double stranded DNA viruses belonging to the family *Poxviridae*, which includes the Sheeppox virus (SPPV), Goatpox virus (GPV) and Lumpy skin disease virus (LSDV) [10]. LSDV is closely related to the two other viruses previously mentioned [11]. Transmission of LSDV is primarily by mechanical means and by several probable arthropod vectors, such as biting flies, mosquitoes (*Aedes aegypti*) [12] and three tick species of the family Ixodidae, namely *Rhipicephalus appendiculatus*, *Rhipicephalus decoloratus* and *Amblyomma hebraeum*. These vectors have been shown to transmit LSDV under experimental conditions, though their capacity to transmit disease under natural field conditions is unknown [10,13].

The morbidity and mortality of the disease vary considerably depending on the breed of cattle, the immunological status of the population and the insect vectors involved in the disease transmission [4]. LSD can cause 1–5% mortality in affected cattle [13]. The severity of LSD has been observed to be higher in *Bos taurus* than in local Zebu (*Bos indicus*). Additionally, lactating cows of either breed are severely affected by LSD [14]. The morbidity can reach as high as 100% in natural outbreaks, while the mortality rate rarely exceeds 5%. Furthermore, during LSD outbreaks, decreased milk production, abortion, infertility, loss of body condition and economic losses have been described [15]. Different approaches can be used as means of prevention and control methods to reduce the disease’s impact. These approaches include vector control and vaccination as the most commonly used options. Four live attenuated *Capripox virus* strains in particular are currently used for vaccine production (Kenyan sheep pox, Yugoslavian RM 65, Romanian sheep pox, and South African Neethling strains). These strains have been used in vaccinations of cattle to protect against LSD infection in different parts of the world due to a major neutralizing site of the *Capripox* virus strains shared by all three strains [14]. However, the control intervention for the 2006 and 2007 LSD epidemics in Israel could not effectively limit the occurrence of LSD after RM65 strain vaccination [16]. Similarly, a questionnaire-based study in Ethiopia showed a lack of efficacy of the LSD vaccine when a massive epidemic occurred among the vaccinated cattle population [17]. Taking this into account, the National Veterinary Institute (NVI) has been working on improving LSD vaccine efficacy. Currently, a live-attenuated vaccine containing the *Capripox* virus strain cultured on VERO– cells is being produced and distributed by NVI to prevent the disease [18].

Epidemiological and vaccine efficacy studies of LSD in Ethiopia have been made based on clinical disease observation and different laboratory tests which have been undertaken in different parts of the country [8,19,20] barring the Sidama regional state. Furthermore, community levels of awareness about LSD disease and vaccination (which could indicate potential prevention measures and interventions) have not been reported. Taking this into consideration, this research was designed to estimate sero-epidemiology and assess community awareness of LSD in terms of their knowledge of the disease as well as its vaccine use and effectiveness, which appeared to be a problem in other parts of the country.

## 2. Material and Methods

### 2.1. Study Area

**Figure 1:**
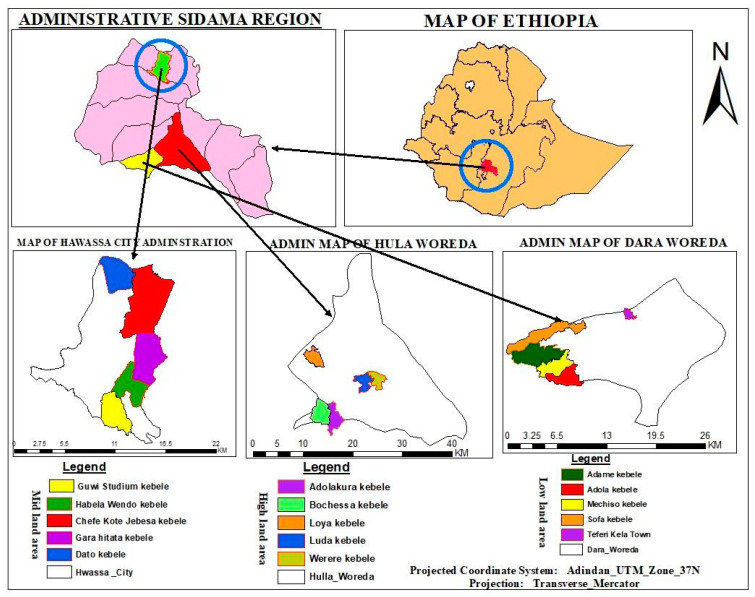
Map of the study area from different administrative hierarchies created by using QGIS 3.36.3.

The study was conducted from September 2021 to June 2022 in the Sidama region, southern Ethiopia. For this purpose, three districts (Hula, Dara and Hawassa) were selected, representing the highland, lowland and midland agro-ecologies, respectively (Figure 1). The agroecological classification of Ethiopia was made based on the Ethiopian Central Statistics Agency (CSA) [21]. Accordingly, altitude, which is <500 m above sea level, is desert, while from 500 to 1300 m above sea level is classified as dry lowland, from 1300 to 2200 m above sea level is classified as midland and from 2200 to 3000 is considered highlands.

The Hula district is 336 km from Addis Ababa and 91 km from Hawassa. Geographically, the district found in 6°29′ N and 38°31′ E and altitude between 2759 and 3000 m.a.s.l. Hula district is a highland district of the region, and the total cattle population in the district is 95,521 [22].

Dara is a district found in the Sidama region that is divided into 37 kebeles (‘Kebele’ is the lowest administrative unit in Ethiopia). Out of these kebeles, six are located in lowland agro-ecology. This district is 365 km from Addis Ababa and 76 km from Hawassa, with 6.47° N and 38.33° E latitude, and it has an altitude that ranges from 1000 to 2500 m.a.s.l as well as a 1200 to 1700 mm mean rain fall [23,24].

Hawassa is the capital city of the Sidama region. Geographically, it lies between 4°27′ and 8°30′ north and 34°21′ and 39°1′ east with an altitude that ranges from 1700 to 2500 m.a.s.l. Hawassa city has a total of eight sub cities and 32 kebeles. The total cattle population in Hawassa is 123568 [23].

### 2.2. Study Population

The target populations were all crossbreed and local breed cattle found in the study areas. Those cattle were mainly managed extensively, while others, mainly in urban and peri-urban areas, were managed semi-intensively. For those cattle, mainly local breeds, management was extensive, with a pasture grazing system being utilized (free-access feeding). However, most of the cross breeds were managed semi-intensively with a majority of either full feeding or supplemental feeding with minimal free-access feeding. The Sidama region is one of the newly formed regions with a huge cattle population estimated to be 2.4 million, according to the report of CSA [24]. The agro-ecological zone and/or district level cattle population estimates were taken from CSA [21,23,25] to know the cattle proportion in the region in correlation with that of the three agro-ecological zones of the region.

Different factors related to the environment and cattle, such as breed, housing system, feeding system, and other management practices, were taken into account and included as potential factors, influencing the disease prevalence.

### 2.3. Study Design and Sample Size Determination

A cross-sectional study design was used in the current study. The minimum sample size for this study was calculated based on predetermined parameters, with a 6.43% expected prevalence (*Pexp*) [19], 95% confidence level (*CI*) and 5% required absolute precision (*d*). The sample size was calculated using the formula described by Thrusfield [26]. The minimum calculated sample size (*n*) for this study was 92.

$$n=\frac{1.96^{2}*Pexp\left(1-Pexp\right)}{d^{2}}$$

*n* = required sample size, *Pexp* = expected prevalence, *d* = desired absolute precision

The minimum calculated sample size was 92; to obtain a representative sample size, it was increased by four-fold (368 cattle). The samples were divided into three representative agro- ecological zones of the region based on the cattle population. The share of cattle to be sampled per district/agro-ecological zone was made proportionally based on the available cattle population from CSA [21,23,25]. Likewise, 31%, 30%, and 39% from low, high, and midland districts were selected, respectively. This share of percentage was found after dividing the total number of cattle in each district by the sum of all the cattle populations in the three districts (agro-ecological zones).

### 2.4. Sampling Method

#### 2.4.1. Animals

A multistage sampling strategy was conducted during sampling techniques involving the use of samples at different hierarchical levels of the aggregated unit of interest. Three districts of the Sidama regional state, representing different agro-ecological zones as well as the cattle population (Hawassa, Dara and Hula), were selected, representing the three agro-ecological zones of the region. Five kebeles were selected from each district/agro-ecological zone based on accessibility and permission from the local authority. A total of 5 herds were selected from each kebele based on the cattle population density. Accordingly, 25 herds were selected from each agro-ecological zone, reaching a total of 75 herds. In this research, a ‘herd’ is defined as a group of cattle that share a common watering and/or feeding sources.

Three agro-ecological zones were represented with kebeles selected from the three different agro-ecological zones. Accordingly, the selected districts representing the different agro-ecological zones include the Dara district, which comprises both midland and lowland areas. The other district representing the midland was the Hawassa City Administration, while the highland agro-ecological part was represented by the Hula district.

Individual samples (blood) were collected following a standard for blood collection [27] from each selected kebele using systematic random selection. Individual and herd prevalence was determined using the results of the serological survey. Different risk factors were assessed for potential correlation with the presence or absence of seroprevalence.

#### 2.4.2. Blood Collection

Blood samples were collected from the jugular vein using disposable plain tubes. All sampled cattle belonged to herds without a history of LSD vaccination. Blood sampling was performed following the principles of good veterinary practice, animal welfare and aseptic methods [28]. Each sample was transported under cold chain conditions to the Hawassa University, Faculty of Veterinary Medicine Microbiology laboratory for serum separation (performed by centrifugation). The separated sera were transferred to sterile cryovials, labeled in tubes with an animal identification number and stored at −20 °C. Finally, all collected sera were shipped to Animal Health Institute (formerly National Animal Health Diagnostic and Investigation, Sebeta) for a virus neutralization test (VNT).

### 2.5. Study Methods

#### 2.5.1. Questionnaire

A questionnaire survey was conducted through face-to-face interviews with 120 individual herd owners and purposefully chosen from three districts. The selected herd owners were individuals who had close relations with cattle. Five kebeles were selected and eight herd owners that agreed to participate in the study were selected from each kebele. This made 40 individual herd owners from each district and a total of 120 individual herd owners from the three districts.

The herd owners/farmers’ abilities to identify LSD infection were cross-checked by enquiring about clinical signs and the local vernacular name for LSD. The description of the disease was necessary in order to avoid confusion with other possible skin diseases, such as dermatophilosis and ringworm. Respondents described the occurrence of a case with clinical signs of generalized skin nodules, fever, peripheral lymph node swelling and ocular/buccal/nasal discharges.

#### 2.5.2. Laboratory Procedures

The virus neutralization test (VNT) was the test of choice performed to detect specific antibodies. The OIE [28] procedures were followed to run the overall process of this laboratory method using 96-wells flat-bottomed tissue culture grade micro-titer plates. Accordingly, each serum was decomplemented by heating at 56 °C for 30 min, and 5-fold dilutions were made using Gibco Minimum Essential Media (MEM) without serum but with 2% antibiotics and antimycotics and 1% glutamine. The virus stock, with a known titer, was also diluted to give 10^3^ TCID50/mL with the same media. The diluted test sera were titrated against a constant titer of *Capripox* virus (100 TCID_50_) 50% tissue culture infective dose in order to calculate a neutralization index. Lamb kidney primary cells were prepared from pre-grown monolayers as a suspension of 4 × 10^5^ cells/mL in a cell culture medium containing antibiotics and 2% fetal calf serum. Following incubation (37 °C) of the micro-titer plates, 100 µL of cell suspension was added to all the wells except the control wells for the medium. The micro-titer plates were covered and incubated at 37 °C for 9 days. Examination of the monolayer cell culture was made starting from days 4 to 9 for the presence of cytopathic effect (CPE). The neutralization index, the log titer difference between the titer of the virus in the negative serum and in the test serum, was used to determine the protective antibody level, considering an index of ≥1.5 as a positive result [28].

### 2.6. Data Analysis

Data collected from qualitative and quantitative studies were entered into a Microsoft Excel spread sheet 2010. The data were coded and filtered before the final data analysis with STATA version 16 software. Descriptive statistics of percentage/prevalence were computed as appropriate to calculate the proportion of the response rate from the questionnaire survey and risk factors in relation to the occurrence of LSD (positive sera for LSD specific antibodies). The difference in the prevalence of LSD, the dependent variable, was analyzed against the independent variables such as sex, age, body condition, breed, management system, and agro-ecological zones using logistic regression analysis with a 95% confidence interval. Odd ratios (OR) were used to associate the statistical strength of LSD positivity with different potential risk factors (independent variables). The level of significance for the statistical test was set at 0.05, which set the significant difference to be considered if found to be *p* < 0.05.

## 3. Results

### 3.1. Questionnaire Survey Result

#### 3.1.1. Farmer Knowledge, Vaccination Habits and Occurrence of LSD in Vaccinated Cattle

From 120 livestock farmers, only 29.2% (35/120) know the disease called “Lumpy skin disease” and have seen the clinical signs and/or heard from animal health professionals. Having knowledge of this disease, 23.3% (28/120) vaccinated their cattle in the past, while 20.8% (25/120) reported the existence of LSD in vaccinated cattle in the same year of vaccination (Table 1).

#### 3.1.2. Respondent’s Demography and Its Association with Farmers’ LSD Knowledge

Respondent’s demography with LSD knowledge happened to be higher proportion in males (30.7%), ≥55 years of age (35.7%) and an educational level of basic writing and reading (34%). However, further analysis of the logistic regression analysis showed the absence of any significant difference between the knowledge of the farmers about LSD and their age, sex, and educational background (*p* > 0.05) (Table 2).

### 3.2. Sero-Epidemology of Lumpy Skin Disease

#### 3.2.1. Animal Level Sero-Prevalence

Out of the total tested samples, 150 sera were found to be positive, with a different level of proportion from 28 to 48% in the three agro-ecological zones. The overall prevalence in the region was 40.8% (Table 3).

#### 3.2.2. Herd Level LSD Sero-Prevalence

Among the 75 herds investigated in the study area, 61 of the herds had at least one sero-positive cattle for LSD. The overall herd level sero-prevalence in study area was 81% (95%CI = 77–85%). The herd level sero-prevalence of LSD was highest in midland (92%) followed by lowland (76%) and highland (76%) (Table 4).

#### 3.2.3. Associated Risk Factors of LSD Occurrence in the Study Area

Logistic regression analysis was performed to assess the association between both animal and environmental risk factors with the LSD serological status. Accordingly, breed and management systems were risk factors found to be significantly associated with LSD sero-positivity (*p* < 0.05). In particular, local breeds of cattle from semi-intensive herds had lower exposure levels of LSD in their serum (Table 5).

## 4. Discussion

The current study addressed the seroprevalence of LSD in three agro-ecological zones of the Sidama region. The overall animal and herd level prevalence of LSD in the present study was 40.8% and 81%, respectively. This individual animal prevalence was in agreement with the previous finding by Welay et al. [2] (41%) from the northern part of Ethiopia. However, Hailu et al. [29] (7.4%), Gari et al. [30] (6%), Abera et al. [19] (6.43%), and Molla et al. [31] (26.5%) reported a lower prevalence than the current one in different parts of Ethiopia. However, a higher prevalence was reported by Albyrak et al. [32] from Turkey (54.6%). These differences could be related to variation in vector population, level of awareness in prevention and control of LSD from different areas. Another factor that can affect the results obtained is the type of test used (ELISA, VNT, etc.) and the diagnostic performance.

The herd-level prevalence of the current finding was higher than the finding of Hailu et al. [29] (44%), which was conducted in north-eastern Ethiopia. The discrepancy in findings may be attributed to the varying research methodologies employed. This study utilized a sero-epidemiological approach, whereas Hailu et al. [29] utilized a questionnaire-based approach to evaluate herd level prevalence, which may increase the likelihood of overlooking undetected cases of LSD by owners.

From the three agro-ecological zones of Sidama regional state, a higher prevalence (48%) was reported in lowland areas followed by midland area (44%). This could be related to the warm and humid climate in mid and lowland agro-climates that favors biting flies, which have their own epidemiological role in LSD transmission [33]. However, there is no significant association between agro-ecological zones with that of LSD occurrence in the study area (*p* > 0.05).

The prevalence of LSD varied significantly among different management systems, and a higher prevalence was recorded in extensive management systems (43%) than semi-intensive management systems (36%). This outcome was in agreement with the findings from other parts of Ethiopia, which were expected to be related to the extensive management production system, which exposes cattle more to the vectors transmitting the disease [31]. Other than the management system risk factors, an animal factor that showed a significant association with the occurrence of LSD was breed (*p* < 0.05). In this study, higher sero-prevalence was observed in cross-bred cattle (73.8%) than local breed cattle (7%). This was in agreement with the findings of Abera et al. [19] in other parts of Ethiopia and Kiplagat et al. [34] from Kenya. This might be related to the difference of innate immune response between the cross and local cattle breed [35].

Vaccination related issues, such as vaccination habits and the practices of livestock farmers were assessed in the study area, which showed that only 23.3% of the livestock farmers vaccinated their cattle against LSD in the past year (from September 2020 to August 2021). In contrast, Gnare et al. [36] reported the use of LSD vaccination by 98.3% of the respondents from the Guto Gida, Wayu Tuka and Gida Ayana districts of western Ethiopia.

Lumpy skin disease is prevalent in Ethiopia, with a different range of prevalence [19,30,31] and an undetermined prevalence in the newly established region, the Sidama regional state. Additionally, LSD-related vaccine efficacy has been studied from different perspectives, mainly questioning the efficacy of the vaccine itself [8,20,37] but not the vaccine delivery from the production site until vaccination. The current study identifies the presence of a knowledge gap about the disease, vaccination habits, and LSD disease occurrence in vaccinated cattle. From 120 interviewed livestock farmers, only 29.2% of them know about LSD. This finding was found to be higher than the study conducted by Moges and Bogale, [38] (11.38%) for north-western Ethiopia. However, the current result was lower than the study performed in East Wollega by Gnare et al. [36] (51.64%). The variation across various regions of the country may be due to either the frequency of cases where livestock farmers starting to familiarized themselves with LSD cases. Both farmers and veterinarians have become more aware of the diseases.

## 5. Conclusions

The results obtained through the questionnaire showed that a smaller proportion of livestock farmers know about LSD (29.2%) and vaccinate their cattle against this disease (23.3%). In addition to the questionnaire survey, sero-epidemiological assessment showed an overall prevalence of 40.8% at animal level in three agro-ecological zones of the Sidama region. From the animal and environmental risk factors analyzed, LSD sero-prevalence showed significant differences between extensive and semi-intensive management systems, as well as between cross-bred and local breeds of cattle in the study area. Hence, an effort to promote awareness among livestock owners to ensure annual cattle vaccination is necessary in the area.

## Figures and Tables

**Table 1:** Qualitative data summary on LSD knowledge and vaccination habits in different districts of the Sidama regional state.

Study Areas		Responses of Farmers Regarding Lumpy Skin Disease (LSD)					
		Do You Know a Disease Called “LSD”		Do You Vaccinate Your Cattle against LSD?		Have You Experienced LSD Disease in Vaccinated Cattle in the Same Year?	
		Yes (%)	No (%)	Yes (%)	No (%)	Yes (%)	No (%)
Hula (*n* = 40)	Werere	3	5	5	3	5	3
	Bochessa	2	6	1	7	1	7
	Adola	2	6	1	7	1	7
	Luda	1	7	0	8	0	8
	Loya	2	6	1	7	1	7
Hawassa (*n* = 40)	Dato	4	4	3	5	1	7
	Guwe	0	8	1	7	2	6
	Gara Hitata	2	6	2	6	0	8
	Chafe	3	5	2	6	0	8
	H/wondo	3	5	0	8	3	5
Dara (*n* = 40)	Millinium	4	4	4	4	2	6
	Safa	2	6	2	6	3	5
	Odole	3	5	2	6	2	6
	Mechisho	2	6	2	6	3	5
	Adame	2	6	2	6	1	7
Over all (N = 120)		35 (29.2)	85 (70.8)	28 (23.3)	92 (76.7)	25 (20.8)	91 (75.8)

**Table 2:** Logistic regression analysis of farmers’ knowledge of LSD and their demographic background (N = 120).

Variables Category		No. of Participant (%)	No. (Proportion) of Participants with the Knowledge of LSD	OR (95%CI)	*p*-Value
Sex	Female	32 (26.7)	8 (25)	Ref.	
	Male	88 (73.3)	27 (30.7)	1.9 (0.7, 5.2)	0.21
Age (in years)	≤35	26 (21.7)	6 (23)	Ref.	
	[36–55)	52 (43.3)	14 (27)	1.3 (0.4, 4.4)	0.72
	≥55	42 (35.0)	15 (35.7)	1.5 (0.4, 5.1)	0.56
Education status	Illiterate	45 (37.50)	12 (26.7)	Ref.	
	Basic writing and reading	41 (34.17)	14 (34)	1.6 (0.6, 4.4)	0.4
	High School	7 (5.83)	1 (14.3)	2.5 (0.2, 26.8)	0.46
	College	27 (22.50)	8 (29.6)	1.3 (0.4, 4.2)	0.64

Ref.: reference.

**Table 3:** Sero-prevalence of LSD in different agro-ecological zones of Sidama regional state (N = 368).

Agro-Ecological Zone	Sampled Cattle	Positive Cattle	Proportion (95%CI)
Highland (Hula)	110	31	28 (20, 37)
Midland (Hawassa)	144	64	44 (36, 47)
Lowland (Dara)	114	55	48 (41, 51)
Overall prevalence	368	150	40.8 (35.8, 45.8)

**Table 4:** Herd Level Prevalence of LSD in Three Agro-Ecological Zones (N = 75).

Agro-Ecological Zone	Sampled Herd Size	Positive Herd Size	Prevalence (%), 95%CI
Highland (Hula)	25	19	76 (66, 82)
Midland (Hawassa)	25	23	92 (84, 94)
Lowland (Dara)	25	19	76 (68, 84)
Overall prevalence	75	61	81 (77, 85)

**Table 5:** Logistic regression analysis of risk factors and the occurrence of LSD sero-positivity.

Risk Factors	No of Sampled	No of Positive	Proportion (%)	COR (95%CI)	AOR (95%CI)	*p*-Values
Sex						
Female	241	107	44.39		Ref.	
Male	127	43	33.85	1.6 (1, 2.44)	2.1 (0.92, 4.61)	0.078
Age						
Young	57	8	14.04		Ref.	
Adult	86	16	18.60	1.4 (0.56, 3.53)	1.1 (0.3, 4)	0.887
Old	225	126	56	7.8 (3.53, 17.22)	2.4 (0.78, 7.2)	0.129
BCS						
Poor	69	36	52.17	1.8 (1.05, 3.1)	Ref.	
Medium	246	93	37.80		1.8 (0.75, 4.5)	0.185
Good	53	21	39.62	1.7 (0.8, 3.4)	3.2 (0.98, 10.43)	0.054
Breed						
Cross	183	135	73.77		Ref.	
Local	185	13	7.03	39.4 (20.5, 75.76)	53 (20.53, 140)	0.000 *
Management systems						
Semi-intensive	127	46	36.22		Ref.	
Extensive	241	104	43.15	1.3 (0.86, 2.1)	3.4 (1.4, 8.7)	0.009 *
Agro-ecological zone						
Highland	110	31	28.18		Ref.	
Lowland	144	55	38.19	2.4 (1.4, 4.1)	1.8 (0.63, 5.03)	0.278
Midland	114	64	56.14	2 (1.2, 3.5)	1.3 (0.5, 3.23)	0.603

*: significant values, AOR = Adjusted OR, COR = crude OR, OR = odd ratio, CI = confidence interval, Ref. = reference.

## Data Availability

Additional information and data can be made available from the corresponding author and principal investigator of this research.
